# Supplementary material for: Estimating the burden of α-thalassaemia in Thailand using a comprehensive prevalence database for Southeast Asia
Source: eLife. 2019 May 23;8:e40580. doi: 10.7554/eLife.40580 (PMC6533055; doi:10.7554/eLife.40580)
Supplement: Supplementary file 1. [file elife-40580-supp1.docx]

## Supplementary file 1

## References for allele frequency data

Sources from which the data points used in the allele maps were identified included:

^1-74^

1. Alauddin H, Langa M, Mohd Yusoff M, et al. Detection of alpha-thalassaemia in neonates on cord blood and dried blood spot samples by capillary electrophoresis. *The Malaysian journal of pathology* 2017; **39**(1): 17-23.

2. Amornkitbamrung S. Screening for Thalassemia Disease, α-thalassemia 1 trait, β-thalassemia trait and Hb E in pregnant women at Nongkhai Hospital. *Chonburi Hospital Journal* 2005; **30**(3): 171-86.

3. Apidechkul T. Prevalence of thalassemia carriers among the Lahu hill tribe population, Chiang Rai, Thailand. *Asian Biomedicine* 2015; **9**(4): 527-33.

4. Ausavarungnirun R, Winichagoon P, Fucharoen S, Epstein N, Simkins R. Detection of zeta-globin chains in the cord blood by ELISA (enzyme-linked immunosorbent assay): Rapid screening for alpha-thalassemia 1 (Southeast Asian type). *Am J Hematol* 1998; **57**(4): 283-6.

5. Chareonkul P, Kraisin J. Prevention and control of thalassemia at Saraburi Regional Hospital. *J Med Assoc Thai* 2004; **1**(8): 8-15.

6. Chinorose S, Toongkam K. Prevalence of Thalassemai Trait and Identification of Couples Risk in Pregnant Women for Severe Thalassemia Disease in Phayao Hospital. *Uttaradit Hospital Medical Bulletin* 2009; **24**(1): 61-9.

7. Dangwibul S, al. e. Efficiency of severe thalassemia screeing program in 5 rural hospitals in Roi Et province (Oral presentation, abstract). The 8th National Thalassemia Academic Symposium. Khon Kaen; 2002. p. 174-5.

8. Fucharoen G, al. e. Thalassemia and iron deficiency in subjects with positive screening OF test and KKU-DCIP-Clear. *Thai J Hematol Transf Med* 1999; **9**: 111-8.

9. Fucharoen G, Sanchaisuriya K, Sae-ung N, Dangwibul S, Fucharoen S. A simplified screening strategy for thalassaemia and haemoglobin E in rural communities in south-east Asia. *Bulletin of the World Health Organization* 2004; **82**(5): 364-72.

10. Fucharoen S, Winichagoon P, Wisedpanichkij R, et al. Prenatal and postnatal diagnoses of thalassemias and hemoglobinopathies by HPLC. *Clin Chem* 1998; **44**(4): 740-8.

11. Hundrieser J, Laig M, Yongvanit P, et al. Study of Alpha-Thalassemia in Northeastern Thailand at the DNA Level. *Hum Hered* 1990; **40**(2): 85-8.

12. Hundrieser J, Sanguansermsri T, Papp T, Flatz G. Alpha-Thalassemia in Northern Thailand - Frequency of Deletional Types Characterized at the DNA Level. *Hum Hered* 1988; **38**(4): 211-5.

13. Jameela S, Sabirah SO, Babam J, et al. Thalassaemia screening among students in a secondary school in Ampang, Malaysia. *The Medical journal of Malaysia* 2011; **66**(5): 522-4.

14. Jearakul W, Khamsaen J. Alpha-thalassemia 1 among Married Couples in Six Northeastern Provinces. *Journal of Health Science* 2009; **18**(5): 728-35.

15. Jindatanmanusan P, Riolueang S, Glomglao W, et al. Diagnostic applications of newborn screening for alpha-thalassaemias, haemoglobins E and H disorders using isoelectric focusing on dry blood spots. *Annals of clinical biochemistry* 2013.

16. Jopang Y. Thalassemia and hemoglobinopathies in anemic schoolchildren The 13th National Thalassemia Academic Symposium. Bangkok; 2007. p. Page 154.

17. Jopang Y. Thalassemia and hemoglobinopathies in anemic schoolchildren. Health Promoting Hospital, Regional Health Promotion Center 5. Nakhonratchasima. *Journal of the Medical Technologist Association of Thailand* 2008; **36**(1): 2235-41.

18. Jopang Y, Mernkratok S, Puangpiruk R. The efficacy of thalassemias and Hb E screening in first-trimester pregnant women at Health Promoting Hospital, Regional Health Promotion Center 5 Nakhonratchasima (poster abstract). The 10th National Thalassemia Academic Symposium. Bangkok; 2004. p. 171.

19. Karakochuk CD, Whitfield KC, Barr SI, et al. Genetic hemoglobin disorders rather than iron deficiency are a major predictor of hemoglobin concentration in women of reproductive age in rural prey veng, Cambodia. *Journal of Nutrition* 2015; **145**(1): 134-42.

20. Karnpean R, Pansuwan A, Fucharoen G, Fucharoen S. Evaluation of the URIT-2900 Automated Hematology Analyzer for screening of thalassemia and hemoglobinopathies in Southeast Asian populations. *Clin Biochem* 2011; **44**(10-11): 889-93.

21. Koh DXR, Raja Sabudin RZA, Mohd Yusoff M, et al. Molecular Characterisation of alpha- and beta-Thalassaemia among Indigenous Senoi Orang Asli Communities in Peninsular Malaysia. *Annals of human genetics* 2017.

22. LemmensZygulska M, Eigel A, Helbig B, Sanguansermsri T, Horst J, Flatz G. Prevalence of alpha-thalassemias in northern Thailand. *Hum Genet* 1996; **98**(3): 345-7.

23. Limsakulsiriratt P, Oncoung W. High Risk Couples for Hb Bart's Hydrops Fetalis in Public Health Region 8 and 9 During 2004-2006. *Chonburi Hospital Journal* 2007; **32**(1): 9-14.

24. Munkongdee T, Pichanun D, Butthep P, et al. Quantitative analysis of Hb Bart's in cord blood by capillary electrophoresis system. *Ann Hematol* 2011; **90**(7): 741-6.

25. Munkongdee T, Tanakulmas J, Butthep P, et al. Molecular Epidemiology of Hemoglobinopathies in Cambodia. *Hemoglobin* 2016; **40**(3): 163-7.

26. Nguyen HV, Sanchaisuriya K, Nguyen D, et al. Thalassemia and Hemoglobinopathies in Thua Thien Hue Province, Central Vietnam. *Hemoglobin* 2013; **37**(4): 333-42.

27. Nguyen NT, Sanchaisuriya K, Sanchaisuriya P, et al. Thalassemia and hemoglobinopathies in an ethnic minority group in Central Vietnam: implications to health burden and relationship between two ethnic minority groups. *Journal of Community Genetics* 2017: 1-8.

28. Nguyen VH, Sanchaisuriya K, Wongprachum K, et al. Hemoglobin Constant Spring is markedly high in women of an ethnic minority group in Vietnam: a community-based survey and hematologic features. *Blood cells, molecules & diseases* 2014; **52**(4): 161-5.

29. Nillakupt K, Nathalang O, Arnutti P, Jindadamrongwech SB, T., Panichkul S, Areekul W. Prevalence and hematological parameters of thalassemia in Tha Kradarn subdistrict Chachoengsao Province, Thailand. *J Med Assoc Thai* 2012; **95**(Suppl 5): S124-S32.

30. O'Riordan S, Hien TT, Miles K, et al. Large scale screening for haemoglobin disorders in southern Vietnam: implications for avoidance and management. *British journal of haematology* 2010; **150**(3): 359-64.

31. Panomai N, Sanchaisuriya K, Yamsri S, et al. Thalassemia and iron deficiency in a group of northeast Thai school children: relationship to the occurrence of anemia. *Eur J Pediatr* 2010; **169**(11): 1317-22.

32. Panyasai S, Cheechang S. The efficiency of screening for carriers of severe thalassemia in three community hospitals in Nakhon Si Thammarat province, Thailand. *Songkla Med J* 2009; **27**(1): 61-72.

33. Panyasai S, Sringam P, Fucharoen G, Sanchaisuriya K, Fucharoen S. A simplified screening for alpha-thalassemia 1 (SEA type) using a combination of a modified osmotic fragility test and a direct PCR on whole blood cell lysates. *Acta Haematol-Basel* 2002; **108**(2): 74-8.

34. Pharephan S, Sirivatanapa P, Makonkawkeyoon S, Tuntiwechapikul W, Makonkawkeyoon L. Prevalence of α-thalassaemia genotypes in pregnant women in northern Thailand. *Indian Journal of Medical Research* 2016; **143**(MARCH): 315-22.

35. Phollarp P, Tritipsombut J, Worasan C, et al. Thalassemia and iron deficiency among pregnant women attending antenatal care service at Khao Wong Hospital, Kalasin province. *Journal of Medical Technology and Physical Therapy* 2010; **22**(3): 262-70.

36. Pichanun D, Munkongdee T, Klamchuen S, et al. MOLECULAR SCREENING OF THE Hbs CONSTANT SPRING (codon 142, TAA > CAA, alpha 2) AND PAKSE (codon 142, TAA > TAT, alpha 2) MUTATIONS IN THAILAND. *Hemoglobin* 2010; **34**(6): 582-6.

37. Rahimah AN, Nisha S, Safiah B, et al. Distribution of alpha thalassaemia in 16 year old Malaysian Students in Penang, Melaka and Sabah. *The Medical journal of Malaysia* 2012; **67**(6): 565-70.

38. Rasri W. Prevention and control of seven thalassemia in pregnancy at Phayao Hospital. *Journal of Health Science* 2008; **17**: 477-84.

39. Rawangkran A, Janwithee N, Wong P, Jermnim N. Prevalence of Thalassemia Trait from Screening Program in Pregnant Women in the Lower Northern Region of Thailand. *Thai J Genet* 2013; **1**: 156-9.

40. Sanchaisuriya K, Fucharoen S, Ratanasiri T, et al. Thalassemia and hemoglobinopathies rather than iron deficiency are major causes of pregnancy-related anemia in northeast Thailand. *Blood Cell Mol Dis* 2006; **37**(1): 8-11.

41. Sangnark P. Prevalence of thalassemia and hemoglobinopathies in pregnant women at Bangkrathum Hospital, Phitsanulok province. *Buddhachinaraj Med J* 2009; **26**(1): 36-43.

42. Sanguansermsri T, Phumyu N, Chomchuen S, Steger HF. Screening for alpha-thalassemia-1 heterozygotes in expecting couples by the combination of a simple erythrocyte osmotic fragility test and a PCR- based method. *Community Genetics* 1999; **2**(1): 26-9.

43. Sanguansermsri T, Steger HF, Sirivatanapa P, Wanapirak C, Tongsong T. Prevention and Control of Severe Thalassemia Syndrome: Chiang Mai strategy. *Thai J Hematol Transf Med* 1998; **8**: 207-14.

44. Sattaratanamai C, Thongsutti S, Sucharitcheep P, Tuengsaeng D, Chomchuen S. Prevalence of Thalassemia and Hemoglobinopathies in Pregnant women at Surin Hospital. *Medical Journal of Srisaket Surin Buriram Hospitals* 2000; **15**(1): 1-12.

45. Savongsy O, Fucharoen S, Fucharoen G, Sanchaisuriya K, Sae-ung N. Thalassemia and hemoglobinopathies in pregnant Lao women: carrier screening, prevalence and molecular basis. *Ann Hematol* 2008; **87**(8): 647-54.

46. Sengchanh S, Sanguansermsri T, Horst D, Horst J, Flatz G. High frequency of alpha-thalassemia in the So ethnic group of south Laos. *Acta Haematol-Basel* 2005; **114**(3): 164-6.

47. Sirichotiyakul S, Tantipalakorn C, Sanguansermsri T, Wanapirak C, Tongsong T. Erythrocyte osmotic fragility test for screening of alpha-thalassemia-1 and beta-thalassemia trait in pregnancy. *Int J Gynecol Obstet* 2004; **86**(3): 347-50.

48. Soonklang M, Nonthalee S, Juntharaniyom M. Thalassemia and hemoglobinopathies in couples, Khon Kaen Hospital (Poster abstract). The 20th National Thalassemia Academic Symposium. Bangkok; 2014. p. Page P29.

49. Sornkayasit K, al. e. Incidence of Hb Constant Spring and Hb Pakse in Khon Kaen: Using capillary electrophoresis and DNA analysis (Poster abstract). The 18th National Thalassemia Academic Symposium. Nonthaburi; 2012. p. 90.

50. Srivorakun H, Fucharoen G, Changtrakul Y, Komwilaisak P, Fucharoen S. Thalassemia and hemoglobinopathies in Southeast Asian newborns: diagnostic assessment using capillary electrophoresis system. *Clin Biochem* 2011; **44**(5-6): 406-11.

51. Sukrat B, Sirichotiyakul S. The prevalence and causes of anemia during pregnancy in Maharaj Nakorn Chiang Mai Hospital. *J Med Assoc Thai 2006 Oct;89 Suppl 4:S142-6* 2006; **89**(Suppl 4): S142-6.

52. Sutjasung P, Fucharoen G, Fucharoen S, Chattumaruk P, Changtrakun D, Sanchaisuriya K. Effectiveness of thalassemia screening with the use of internal quality control blood samples at Kasetsomboon Hospital, Chaiyaphoom province. *JOURNAL OF MEDICAL TECHNOLOGY AND PHYSICAL THERAPY* 2011; **23**(1): 34-45.

53. Suwannakhon N, Seeratanachot T, Mahingsa K, Namwong P, T. S. Prevalence of Alpha-thalassemia Trait in the Volunteered Personals of University of Phayao. *J Hematol Transfus Med* 2014; **24**: 129-36.

54. Tan JA, Tay JS, Soemantri A, et al. Deletional types of alpha-thalassaemia in central Java. *Hum Hered* 1992; **42**(5): 289-92.

55. Tan JAMA, Lee PC, Wee YC, et al. High prevalence of alpha- and beta-thalassemia in the kadazandusuns in east Malaysia: Challenges in providing effective health care for an indigenous group. *Journal of Biomedicine and Biotechnology* 2010; **2010**.

56. Tangvarasittichai O, Jeenapongsa R, Sitthiworanan C, Sanguansermsri T. Laboratory investigations of Hb Constant Spring. *Clin Lab Haematol* 2005; **27**(1): 47-9.

57. Tangvarasittichai O, Poonanan N, Tangvarasittichai S. Using Red Cell Indices and Reticulocyte Parameters for Carrier Screening of Various Thalassemia Syndromes. *Indian journal of clinical biochemistry : IJCB* 2017; **32**(1): 61-7.

58. Tanphaichitr VS, Pung-amritt P, Puchaiwatananon O, et al. Studies on hemoglobin Bart's and deletion of alpha-globin genes from cord blood in Thailand (poster abstract). The International Conference on Thalassemia. Bangkok; 1985. p. P05.

59. Than AM, Harano T, Harano K, Myint AA, Ogino T, Okada S. High incidence of alpha-thalassemia, hemoglobin E, and glucose-6-phosphate dehydrogenase deficiency in populations of malaria-endemic southern Shan State, Myanmar. *Int J Hematol* 2005; **82**(2): 119-23.

60. Thanomrat P, Wannapira W, Sritippawan S, Boon-eam O. The prevalence of alpha-thalassemia trait in Buddhachinaraj Phitsanulok Hospital preliminary report. *Buddhachinaraj Med J* 2003; **20**(1): 19-25.

61. Thongon R, al. e. Strategy for thalassemia screening at Yala hospital (Poster abstract). The 15 th National Thalassemia Academic Symposium. Udon Thani; 2009. p. 144.

62. Tienthavorn V, Pattanapongsthorn J, Charoensak S, Sae-Tung R, Charoenkwan P, Sanguansermsri T. Prevalence of Thalassemia Carriers in Thailand. *Thai J Hematol Transf Med* 2006; **16**: 307-12.

63. Tongon R, Yunu R, Sanchaisuriya K, et al. Thalassemia and hemoglobinopathies in pregnant women attended antenatal care service at Yala Hospital. *J Med Tech Phy Ther* 2014; **26**(1): 32-9.

64. Traisrisilp K, Jatavan P, Tongsong T. A retrospective comparison of pregnancy outcomes between women with alpha-thalassaemia 1 trait and normal controls. *Journal of obstetrics and gynaecology : the journal of the Institute of Obstetrics and Gynaecology* 2017: 1-4.

65. Trisakul N, Siripulsak P, ​Chuesupalobol W. Prevalence ​of​ Thalassemia,​ Hemoglobinopathy,​ At-Risk ​Couples​ and​ Incidence ​of ​Thalassemia ​Major ​from​ the ​Screening ​Program, Prenatal ​and ​Postnatal ​Diagnosis​ at ​Yasothorn ​Hospital. *J Hematol Transfus Med* 2008; **19**: 285-92.

66. Tritipsombut J, Sanchaisuriya K, Fucharoen S, et al. Hemoglobin Profiles and Hematologic Features of Thalassemic Newborns Application to Screening of alpha-Thalassemia 1 and Hemoglobin E. *Arch Pathol Lab Med* 2008; **132**(11): 1739-45.

67. Tritipsombut J, Sanchaisuriya K, Phollarp P, et al. Micromapping of Thalassemia and Hemoglobinopathies in Diferent Regions of Northeast Thailand and Vientaine, Laos People's Democratic Republic. *Hemoglobin* 2012; **36**(1): 47-56.

68. Uaprasert N, Settapiboon R, Amornsiriwat S, et al. Diagnostic utility of isoelectric focusing and high performance liquid chromatography in neonatal cord blood screening for thalassemia and non-sickling hemoglobinopathies. *Clinica chimica acta; international journal of clinical chemistry* 2014; **427**: 23-6.

69. Wanapirak C, Muninthorn W, Sanguansermsri T, Dhananjayanonda P, Tongsong T. Prevalence of Thalassemia in pregnant women at Maharaj Nakorn Chiang Mai Hospital. *Journal of the Medical Association of Thailand* 2004; **87**(12): 1415-8.

70. Wong P, Thanormrat P, Srithipayawan S, et al. Risk of a couple having a child with severe thalassemia syndrome, prevalence in lower northern Thailand. *Southeast Asian Journal of Tropical Medicine and Public Health* 2006; **37**(2): 366-9.

71. Wong P, Thanormrat P, Srithipayawan S, et al. Prevalence of thalassemia trait from screening program in pregnant women of Phitsanulok. *Thai J Hematol Transf Med* 2004; **14**: 181-6.

72. Wongkham J, Ratanasiri T, Komwilaisak R, Saksiriwuttho P, Paibool M, Chatvised P. Thalassemia screening in pregnant women at antenatal care clinic, Srinagarind Hospital. *Srinagarind Med J* 2013; **28**(2): 170-7.

73. Yap ZM, Sun KM, Teo CRL, Tan ASC, Chong SS. Evidence of differential selection for the -alpha(3.7) and -alpha(4.2) single-alpha-globin gene deletions within the same population. *Eur J Haematol* 2013; **90**(3): 210-3.

74. Yin SKK, Chong QT, Mei LA, et al. A molecular epidemiologic study of thalassemia using newborns' cord blood in a multiracial Asian population in Singapore - Results and recommendations for a population screening program. *J Pediat Hematol Onc* 2004; **26**(12): 817-9.
