# Supplementary material for: Estimating the burden of α-thalassaemia in Thailand using a comprehensive prevalence database for Southeast Asia
Source: eLife. 2019 May 23;8:e40580. doi: 10.7554/eLife.40580 (PMC6533055; doi:10.7554/eLife.40580)
Supplement: Supplementary file 2. [file elife-40580-supp2.docx]

## Supplementary file 2

## References for genetic variant data

Sources from which the data points used in the genetic variation maps were identified included:

^1-47^

1. Alauddin H, Langa M, Mohd Yusoff M, et al. Detection of alpha-thalassaemia in neonates on cord blood and dried blood spot samples by capillary electrophoresis. *The Malaysian journal of pathology* 2017; **39**(1): 17-23.

2. Fucharoen G, al. e. Thalassemia and iron deficiency in subjects with positive screening OF test and KKU-DCIP-Clear. *Thai J Hematol Transf Med* 1999; **9**: 111-8.

3. Fucharoen G, Sanchaisuriya K, Sae-ung N, Dangwibul S, Fucharoen S. A simplified screening strategy for thalassaemia and haemoglobin E in rural communities in south-east Asia. *Bulletin of the World Health Organization* 2004; **82**(5): 364-72.

4. Fucharoen S, Winichagoon P, Wisedpanichkij R, et al. Prenatal and postnatal diagnoses of thalassemias and hemoglobinopathies by HPLC. *Clin Chem* 1998; **44**(4): 740-8.

5. Hundrieser J, Laig M, Yongvanit P, et al. Study of Alpha-Thalassemia in Northeastern Thailand at the DNA Level. *Hum Hered* 1990; **40**(2): 85-8.

6. Hundrieser J, Sanguansermsri T, Papp T, Flatz G. Alpha-Thalassemia in Northern Thailand - Frequency of Deletional Types Characterized at the DNA Level. *Hum Hered* 1988; **38**(4): 211-5.

7. Jameela S, Sabirah SO, Babam J, et al. Thalassaemia screening among students in a secondary school in Ampang, Malaysia. *The Medical journal of Malaysia* 2011; **66**(5): 522-4.

8. Jearakul W, Khamsaen J. Alpha-thalassemia 1 among Married Couples in Six Northeastern Provinces. *Journal of Health Science* 2009; **18**(5): 728-35.

9. Jindatanmanusan P, Riolueang S, Glomglao W, et al. Diagnostic applications of newborn screening for alpha-thalassaemias, haemoglobins E and H disorders using isoelectric focusing on dry blood spots. *Annals of clinical biochemistry* 2013.

10. Karakochuk CD, Whitfield KC, Barr SI, et al. Genetic hemoglobin disorders rather than iron deficiency are a major predictor of hemoglobin concentration in women of reproductive age in rural prey veng, Cambodia. *Journal of Nutrition* 2015; **145**(1): 134-42.

11. Karnpean R, Pansuwan A, Fucharoen G, Fucharoen S. Evaluation of the URIT-2900 Automated Hematology Analyzer for screening of thalassemia and hemoglobinopathies in Southeast Asian populations. *Clin Biochem* 2011; **44**(10-11): 889-93.

12. Koh DXR, Raja Sabudin RZA, Mohd Yusoff M, et al. Molecular Characterisation of alpha- and beta-Thalassaemia among Indigenous Senoi Orang Asli Communities in Peninsular Malaysia. *Annals of human genetics* 2017.

13. LemmensZygulska M, Eigel A, Helbig B, Sanguansermsri T, Horst J, Flatz G. Prevalence of alpha-thalassemias in northern Thailand. *Hum Genet* 1996; **98**(3): 345-7.

14. Limsakulsiriratt P, Oncoung W. High Risk Couples for Hb Bart's Hydrops Fetalis in Public Health Region 8 and 9 During 2004-2006. *Chonburi Hospital Journal* 2007; **32**(1): 9-14.

15. Munkongdee T, Pichanun D, Butthep P, et al. Quantitative analysis of Hb Bart's in cord blood by capillary electrophoresis system. *Ann Hematol* 2011; **90**(7): 741-6.

16. Munkongdee T, Tanakulmas J, Butthep P, et al. Molecular Epidemiology of Hemoglobinopathies in Cambodia. *Hemoglobin* 2016; **40**(3): 163-7.

17. Nguyen HV, Sanchaisuriya K, Nguyen D, et al. Thalassemia and Hemoglobinopathies in Thua Thien Hue Province, Central Vietnam. *Hemoglobin* 2013; **37**(4): 333-42.

18. Nguyen NT, Sanchaisuriya K, Sanchaisuriya P, et al. Thalassemia and hemoglobinopathies in an ethnic minority group in Central Vietnam: implications to health burden and relationship between two ethnic minority groups. *Journal of Community Genetics* 2017: 1-8.

19. Nguyen VH, Sanchaisuriya K, Wongprachum K, et al. Hemoglobin Constant Spring is markedly high in women of an ethnic minority group in Vietnam: a community-based survey and hematologic features. *Blood cells, molecules & diseases* 2014; **52**(4): 161-5.

20. Nillakupt K, Nathalang O, Arnutti P, Jindadamrongwech SB, T., Panichkul S, Areekul W. Prevalence and hematological parameters of thalassemia in Tha Kradarn subdistrict Chachoengsao Province, Thailand. *J Med Assoc Thai* 2012; **95**(Suppl 5): S124-S32.

21. O'Riordan S, Hien TT, Miles K, et al. Large scale screening for haemoglobin disorders in southern Vietnam: implications for avoidance and management. *British journal of haematology* 2010; **150**(3): 359-64.

22. Panomai N, Sanchaisuriya K, Yamsri S, et al. Thalassemia and iron deficiency in a group of northeast Thai school children: relationship to the occurrence of anemia. *Eur J Pediatr* 2010; **169**(11): 1317-22.

23. Panyasai S, Cheechang S. The efficiency of screening for carriers of severe thalassemia in three community hospitals in Nakhon Si Thammarat province, Thailand. *Songkla Med J* 2009; **27**(1): 61-72.

24. Pharephan S, Sirivatanapa P, Makonkawkeyoon S, Tuntiwechapikul W, Makonkawkeyoon L. Prevalence of α-thalassaemia genotypes in pregnant women in northern Thailand. *Indian Journal of Medical Research* 2016; **143**(MARCH): 315-22.

25. Phollarp P, Tritipsombut J, Worasan C, et al. Thalassemia and iron deficiency among pregnant women attending antenatal care service at Khao Wong Hospital, Kalasin province. *Journal of Medical Technology and Physical Therapy* 2010; **22**(3): 262-70.

26. Pichanun D, Munkongdee T, Klamchuen S, et al. MOLECULAR SCREENING OF THE Hbs CONSTANT SPRING (codon 142, TAA > CAA, alpha 2) AND PAKSE (codon 142, TAA > TAT, alpha 2) MUTATIONS IN THAILAND. *Hemoglobin* 2010; **34**(6): 582-6.

27. Rahimah AN, Nisha S, Safiah B, et al. Distribution of alpha thalassaemia in 16 year old Malaysian Students in Penang, Melaka and Sabah. *The Medical journal of Malaysia* 2012; **67**(6): 565-70.

28. Sanchaisuriya K, Fucharoen S, Ratanasiri T, et al. Thalassemia and hemoglobinopathies rather than iron deficiency are major causes of pregnancy-related anemia in northeast Thailand. *Blood Cell Mol Dis* 2006; **37**(1): 8-11.

29. Savongsy O, Fucharoen S, Fucharoen G, Sanchaisuriya K, Sae-ung N. Thalassemia and hemoglobinopathies in pregnant Lao women: carrier screening, prevalence and molecular basis. *Ann Hematol* 2008; **87**(8): 647-54.

30. Sengchanh S, Sanguansermsri T, Horst D, Horst J, Flatz G. High frequency of alpha-thalassemia in the So ethnic group of south Laos. *Acta Haematol-Basel* 2005; **114**(3): 164-6.

31. Setianingsih I, Harahap A, Nainggolan IM. Alpha thalassaemia in Indonesia: phenotypes and molecular defects. *Advances in experimental medicine and biology* 2003; **531**: 47-56.

32. Sornkayasit K, al. e. Incidence of Hb Constant Spring and Hb Pakse in Khon Kaen: Using capillary electrophoresis and DNA analysis (Poster abstract). The 18th National Thalassemia Academic Symposium. Nonthaburi; 2012. p. 90.

33. Srivorakun H, Fucharoen G, Changtrakul Y, Komwilaisak P, Fucharoen S. Thalassemia and hemoglobinopathies in Southeast Asian newborns: diagnostic assessment using capillary electrophoresis system. *Clin Biochem* 2011; **44**(5-6): 406-11.

34. Sutjasung P, Fucharoen G, Fucharoen S, Chattumaruk P, Changtrakun D, Sanchaisuriya K. Effectiveness of thalassemia screening with the use of internal quality control blood samples at Kasetsomboon Hospital, Chaiyaphoom province. *JOURNAL OF MEDICAL TECHNOLOGY AND PHYSICAL THERAPY* 2011; **23**(1): 34-45.

35. Suwannakhon N, Seeratanachot T, Mahingsa K, Namwong P, T. S. Prevalence of Alpha-thalassemia Trait in the Volunteered Personals of University of Phayao. *J Hematol Transfus Med* 2014; **24**: 129-36.

36. Tan JA, Tay JS, Soemantri A, et al. Deletional types of alpha-thalassaemia in central Java. *Hum Hered* 1992; **42**(5): 289-92.

37. Tan JAMA, Lee PC, Wee YC, et al. High prevalence of alpha- and beta-thalassemia in the kadazandusuns in east Malaysia: Challenges in providing effective health care for an indigenous group. *Journal of Biomedicine and Biotechnology* 2010; **2010**.

38. Tangvarasittichai O, Jeenapongsa R, Sitthiworanan C, Sanguansermsri T. Laboratory investigations of Hb Constant Spring. *Clin Lab Haematol* 2005; **27**(1): 47-9.

39. Tangvarasittichai O, Poonanan N, Tangvarasittichai S. Using Red Cell Indices and Reticulocyte Parameters for Carrier Screening of Various Thalassemia Syndromes. *Indian journal of clinical biochemistry : IJCB* 2017; **32**(1): 61-7.

40. Tanphaichitr VS, Pung-amritt P, Puchaiwatananon O, et al. Studies on hemoglobin Bart's and deletion of alpha-globin genes from cord blood in Thailand (poster abstract). The International Conference on Thalassemia. Bangkok; 1985. p. P05.

41. Than AM, Harano T, Harano K, Myint AA, Ogino T, Okada S. High incidence of alpha-thalassemia, hemoglobin E, and glucose-6-phosphate dehydrogenase deficiency in populations of malaria-endemic southern Shan State, Myanmar. *Int J Hematol* 2005; **82**(2): 119-23.

42. Tongon R, Yunu R, Sanchaisuriya K, et al. Thalassemia and hemoglobinopathies in pregnant women attended antenatal care service at Yala Hospital. *J Med Tech Phy Ther* 2014; **26**(1): 32-9.

43. Tritipsombut J, Sanchaisuriya K, Fucharoen S, et al. Hemoglobin Profiles and Hematologic Features of Thalassemic Newborns Application to Screening of alpha-Thalassemia 1 and Hemoglobin E. *Arch Pathol Lab Med* 2008; **132**(11): 1739-45.

44. Tritipsombut J, Sanchaisuriya K, Phollarp P, et al. Micromapping of Thalassemia and Hemoglobinopathies in Diferent Regions of Northeast Thailand and Vientaine, Laos People's Democratic Republic. *Hemoglobin* 2012; **36**(1): 47-56.

45. Uaprasert N, Settapiboon R, Amornsiriwat S, et al. Diagnostic utility of isoelectric focusing and high performance liquid chromatography in neonatal cord blood screening for thalassemia and non-sickling hemoglobinopathies. *Clinica chimica acta; international journal of clinical chemistry* 2014; **427**: 23-6.

46. Yap ZM, Sun KM, Teo CRL, Tan ASC, Chong SS. Evidence of differential selection for the -alpha(3.7) and -alpha(4.2) single-alpha-globin gene deletions within the same population. *Eur J Haematol* 2013; **90**(3): 210-3.

47. Yin SKK, Chong QT, Mei LA, et al. A molecular epidemiologic study of thalassemia using newborns' cord blood in a multiracial Asian population in Singapore - Results and recommendations for a population screening program. *J Pediat Hematol Onc* 2004; **26**(12): 817-9.
