# Supplementary material for: Estimating the burden of α-thalassaemia in Thailand using a comprehensive prevalence database for Southeast Asia
Source: eLife. 2019 May 23;8:e40580. doi: 10.7554/eLife.40580 (PMC6533055; doi:10.7554/eLife.40580)
Supplement: Transparent reporting form [file elife-40580-transrepform.docx]

***eLife’s* transparent reporting form**

We encourage authors to provide detailed information *within their submission* to facilitate the interpretation and replication of experiments. Authors can upload supporting documentation to indicate the use of appropriate reporting guidelines for health-related research (see [EQUATOR Network](http://www.equator-network.org/%20)), life science research (see the [BioSharing Information Resource](https://biosharing.org/)), or the [ARRIVE guidelines](http://www.plosbiology.org/article/info:doi/10.1371/journal.pbio.1000412) for reporting work involving animal research. Where applicable, authors should refer to any relevant reporting standards documents in this form.

If you have any questions, please consult our Journal Policies and/or contact us: [editorial@elifesciences.org](mailto:editorial@elifesciences.org).

**Sample-size estimation**

- You should state whether an appropriate sample size was computed when the study was being designed
- You should state the statistical method of sample size computation and any required assumptions
- If no explicit power analysis was used, you should describe how you decided what sample (replicate) size (number) to use

Please outline where this information can be found within the submission (e.g., sections or figure legends), or explain why this information doesn’t apply to your submission:

This study used pre-existing data that was available in published literature. As a result, no sample size was computed. The literature review was undertaken using a rigorous inclusion/exclusion protocol to identify suitable data sources for the study.

**Replicates**

- You should report how often each experiment was performed
- You should include a definition of biological versus technical replication
- The data obtained should be provided and sufficient information should be provided to indicate the number of independent biological and/or technical replicates
- If you encountered any outliers, you should describe how these were handled
- Criteria for exclusion/inclusion of data should be clearly stated
- High-throughput sequence data should be uploaded before submission, with a private link for reviewers provided (these are available from both GEO and ArrayExpress)

Please outline where this information can be found within the submission (e.g., sections or figure legends), or explain why this information doesn’t apply to your submission:

This is not applicable to the present study.

**Statistical reporting**

- Statistical analysis methods should be described and justified
- Raw data should be presented in figures whenever informative to do so (typically when N per group is less than 10)
- For each experiment, you should identify the statistical tests used, exact values of N, definitions of center, methods of multiple test correction, and dispersion and precision measures (e.g., mean, median, SD, SEM, confidence intervals; and, for the major substantive results, a measure of effect size (e.g., Pearson's r, Cohen's d)
- Report exact p-values wherever possible alongside the summary statistics and 95% confidence intervals. These should be reported for all key questions and not only when the p-value is less than 0.05.

Please outline where this information can be found within the submission (e.g., sections or figure legends), or explain why this information doesn’t apply to your submission:

### The statistical analysis methods are outlined in the Methods section under the sub-heading “Modelling continuous maps of α-thalassaemia allele frequency in Thailand” and described in detail in the Supplementary methods 2 section. In brief, we used a Bayesian geostatistical modelling framework based on the Integrated Nested Laplace Approximation (INLA) package implemented in R.

### The total number of data points for each of the three geostatistical models used to generate the continuous maps of the different α-thalassaemia forms in Thailand are written in the main text, under the Results section sub-heading “Continuous allele frequency maps for Thailand”. Mean, median and uncertainty summary maps are presented throughout the study.

The 95% credible intervals for our newborn estimates are provided in the text whilst maps of the 95% credible intervals for our frequency estimates are provided in Figure 1. We have made it clear in the text that the mean of the posterior predictive distribution is reported.

The other sections of the paper are descriptive and therefore do not involve any statistical testing.

(For large datasets, or papers with a very large number of statistical tests, you may upload a single table file with tests, Ns, etc., with reference to sections in the manuscript.)

**Group allocation**

- Indicate how samples were allocated into experimental groups (in the case of clinical studies, please specify allocation to treatment method); if randomization was used, please also state if restricted randomization was applied
- Indicate if masking was used during group allocation, data collection and/or data analysis

Please outline where this information can be found within the submission (e.g., sections or figure legends), or explain why this information doesn’t apply to your submission:

This is not applicable to the present study.

**Additional data files (“source data”)**

- We encourage you to upload relevant additional data files, such as numerical data that are represented as a graph in a figure, or as a summary table
- Where provided, these should be in the most useful format, and they can be uploaded as “Source data” files linked to a main figure or table
- Include model definition files including the full list of parameters used
- Include code used for data analysis (e.g., R, MatLab)
- Avoid stating that data files are “available upon request”

Please indicate the figures or tables for which source data files have been provided:

Figure 1 displays the predicted allele frequencies that were obtained from our geostatistical model. As the included maps have a resolution of 1km x 1km, the data that underpin the maps are large and also meaningless in table format. The raw data that were used to generate the predictive model are presented in Figure 2 and are provided in the *Supplementary data* (“Figure-2A Source data”, “Figure-2B Source data”, “Figure-2C Source data”). Please note that only a subset of these data were used to generate the model (those that come from Thailand and its neighbouring countries).

The data subset used to construct the map in Figure 3 is provided in the *Supplementary data* (“Figure-3 Source data”).

The data subsets used to construct the three maps in Figures 4-6 are provided in the *Supplementary data* (“Figure-4-5-6 Source data”).

The dataset used to construct Supplementary figure 2 are provided in the *Supplementary data* (“Supplementary figure-2 Source data”).
